# Intraoperative patient‐specific volumetric reconstruction and 3D visualization for laparoscopic liver surgery

**DOI:** 10.1049/htl2.12106

**Published:** 2024-12-09

**Authors:** Luca Boretto, Egidijus Pelanis, Alois Regensburger, Kaloian Petkov, Rafael Palomar, Åsmund Avdem Fretland, Bjørn Edwin, Ole Jakob Elle

**Affiliations:** ^1^ Siemens Healthcare AS Oslo Norway; ^2^ Department of Informatics Faculty of Mathematics and Natural Sciences University of Oslo Oslo Norway; ^3^ The Intervention Centre Oslo University Hospital Rikshospitalet Oslo Norway; ^4^ Siemens Healthineers AG Forchheim Germany; ^5^ Siemens Medical Solutions USA, Inc. Princeton New Jersey USA; ^6^ Department of Computer Science Norwegian University of Science and Technology Gjøvik Norway; ^7^ Department of HPB Surgery Oslo University Hospital Rikshospitalet Oslo Norway; ^8^ Faculty of Medicine Institute of Medicine University of Oslo Oslo Norway

**Keywords:** augmented reality, biomedical imaging, computer vision, liver, medical image processing, object tracking, optical tracking, ultrasonic imaging, virtual reality

## Abstract

Despite the benefits of minimally invasive surgery, interventions such as laparoscopic liver surgery present unique challenges, like the significant anatomical differences between preoperative images and intraoperative scenes due to pneumoperitoneum, patient pose, and organ manipulation by surgical instruments. To address these challenges, a method for intraoperative three‐dimensional reconstruction of the surgical scene, including vessels and tumors, without altering the surgical workflow, is proposed. The technique combines neural radiance field reconstructions from tracked laparoscopic videos with ultrasound three‐dimensional compounding. The accuracy of our reconstructions on a clinical laparoscopic liver ablation dataset, consisting of laparoscope and patient reference posed from optical tracking, laparoscopic and ultrasound videos, as well as preoperative and intraoperative computed tomographies, is evaluated. The authors propose a solution to compensate for liver deformations due to pressure applied during ultrasound acquisitions, improving the overall accuracy of the three‐dimensional reconstructions compared to the ground truth intraoperative computed tomography with pneumoperitoneum. A unified neural radiance field from the ultrasound and laparoscope data, which allows real‐time view synthesis providing surgeons with comprehensive intraoperative visual information for laparoscopic liver surgery, is trained.

## Graphical abstract

A method for intraoperative three‐dimensional reconstruction in laparoscopic liver surgery by integrating neural radiance field reconstructions from tracked laparoscopic videos with three‐dimensional ultrasound compounding is proposed. This approach addresses anatomical changes caused by pneumoperitoneum and ultrasound probe pressure, providing surgeons with photorealistic visualizations by training a unified neural radiance field from both ultrasound and laparoscopic data. Evaluation on a clinical dataset shows high accuracy in three‐dimensional reconstructions compared to ground truth intraoperative computed tomography.

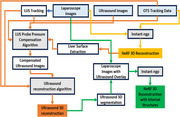

## 1 INTRODUCTION

Laparoscopic surgery has become the gold standard for minimally invasive liver tumor resections, offering numerous advantages over traditional open surgery, including reduced post‐surgical pain, faster recovery times, and improved cosmetic outcomes [1, 2, 3, 4]. However, laparoscopic procedures present unique challenges. During laparoscopic surgery, the abdominal cavity is insufflated with CO_2_ (pneumoperitoneum), leading to the deformation of organs; in addition, the patient is positioned differently compared to the preoperative imaging stage. Consequently, the intraoperative liver anatomy, altered by pneumoperitoneum and gravity, differs significantly from preoperative images. Surgeons rely on laparoscopic video to visualize the intra‐abdominal cavity and B‐mode images from a laparoscopic ultrasound (LUS) probe to orient themselves and locate vessels and tumors, requiring them to mentally correlate intraoperative images with preoperative volumes. This process can be particularly challenging for complex procedures, especially those involving liver mobilization, due to organ deformations, a limited surgical field of view, and variability in surgical skill.

Moreover, the organ's position, orientation, and deformation state change multiple times during the procedure, making a single intraoperative CT scan with pneumoperitoneum insufficient and impractical. Furthermore, CT scanners are not standard in operating theaters. To address these limitations, preclinical and clinical research has investigated various surgical navigation solutions. Several groups have examined the use of biomechanical models aligned with intraoperative scenes utilizing laparoscopic video or ultrasound [5, 6, 7]. Researchers have also explored the use of three‐dimensional (3D) reconstructions from both ultrasound data and laparoscopic videos. Intraoperative ultrasound (IOUS) provides a real‐time visualization of the liver parenchyma, aiding in the identification of tumors and vascular structures. However, IOUS images are challenging to interpret due to their 2D nature and limited field of view. Various groups have investigated ultrasound 3D reconstruction [8, 9, 10, 11, 12]. However, the contact pressure the surgeon applies during ultrasound acquisition can deform the organ, altering the location of vessels and tumors in the images, thus compromising the resulting 3D reconstruction [8, 13]. Some research efforts have focused on compensating for this probe pressure; however, they assume the availability of an intraoperative CT or prior knowledge of the biomechanical properties of the organ [13], which differ from patient to patient, or perform relative image‐based deformation [12], leading to consistent reconstructions but incorrect coordinate systems. Boretto et al. [14] established the accuracy of a hybrid tracking approach for LUS probes, combining optical tracking with laparoscopic image‐based tracking. This approach offers an accurate and reliable method for tracking the ultrasound probe's position within the surgical field, enabling this tracking information to perform ultrasound 3D reconstructions of the organ's internal structures. Several groups have explored reconstructing the liver surface from laparoscopic images [6, 15–18]. Many use traditional methods like simultaneous localization and mapping [19, 20, 21] relying on feature extraction and matching, which can be prone to errors when used in an environment lacking visual features like the liver surface. Alternatively, neural radiance fields (NeRFs) do not rely on extracting explicit features from images to reconstruct a scene. NeRF implementations have been investigated previously in laparoscopic surgical videos, showing promising results [22, 23]. However, although 3D reconstructions from laparoscopic videos provide valuable insights into the overall liver surface, they lack information about the organ's internal structures. To the best of our knowledge, no published work has demonstrated on a clinical dataset the integration of ultrasound and laparoscopic information into a single, unified 3D reconstruction within the same coordinate system.

Our contribution includes the following:

- Integrating 3D reconstructions from laparoscopic images and ultrasound into a common optical tracker coordinate system.
- Compensating for the pressure applied by the LUS probe using the 3D reconstruction from the tracked laparoscope.
- Evaluating, as a proof of concept, the accuracy of the resulting reconstructions obtained with this method using a clinical dataset, with intraoperative CT serving as the ground truth (GT).
- Evaluating the quality of NeRF reconstructions from laparoscopic images tracked using an optical tracking system (OTS).
- Introducing a novel approach to simultaneously visualize the abdominal cavity and internal organ structures obtained via ultrasound, utilizing NeRFs.

The aim of this work is to develop a workflow that enables accurate surgical navigation through an intraoperative 3D reconstruction of the liver, without altering the surgical workflow except for the optical tracking of the laparoscope.

## 2 METHODS

In this study, we utilize OTS data, along with laparoscope and ultrasound (LUS) images, to generate comprehensive 3D reconstructions of the liver during laparoscopic surgery.

**FIGURE 1:**
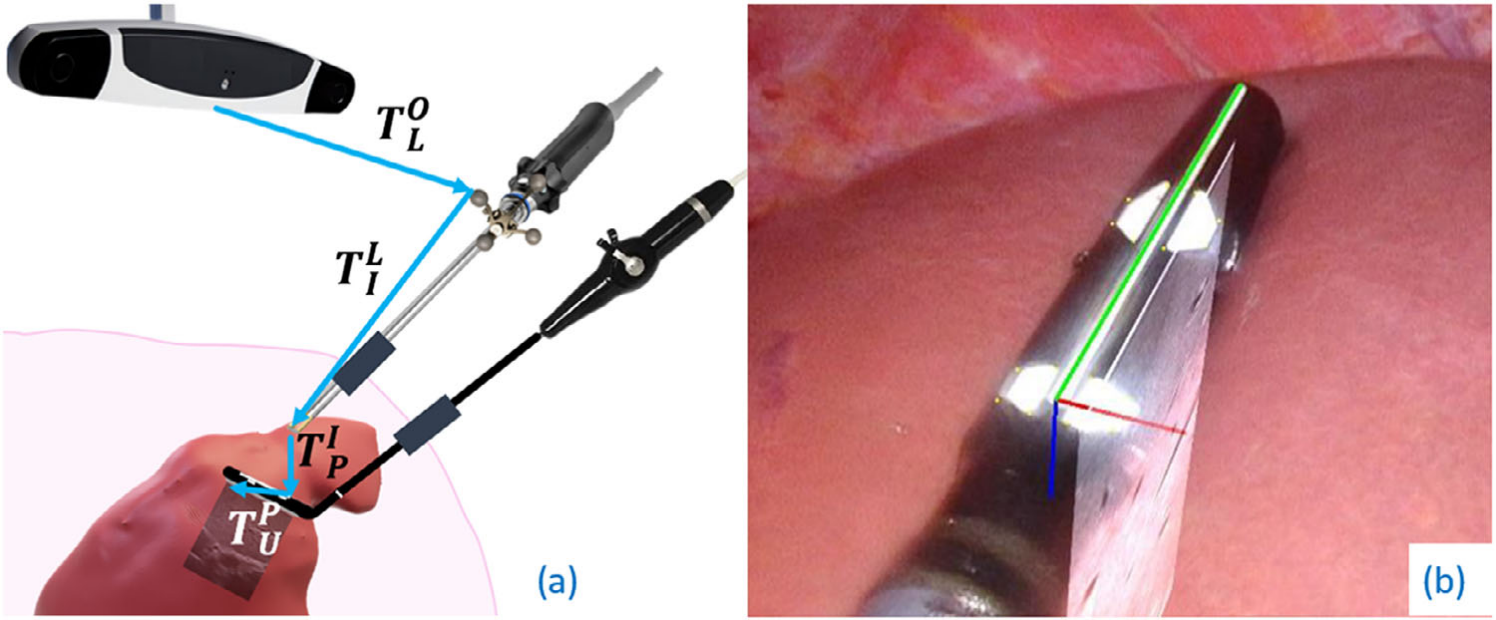
(a) Clinical acquisition setup and chain of transformations to track the LUS probe images. (b) LUS probe tracking from laparoscope image.

**FIGURE 2:**
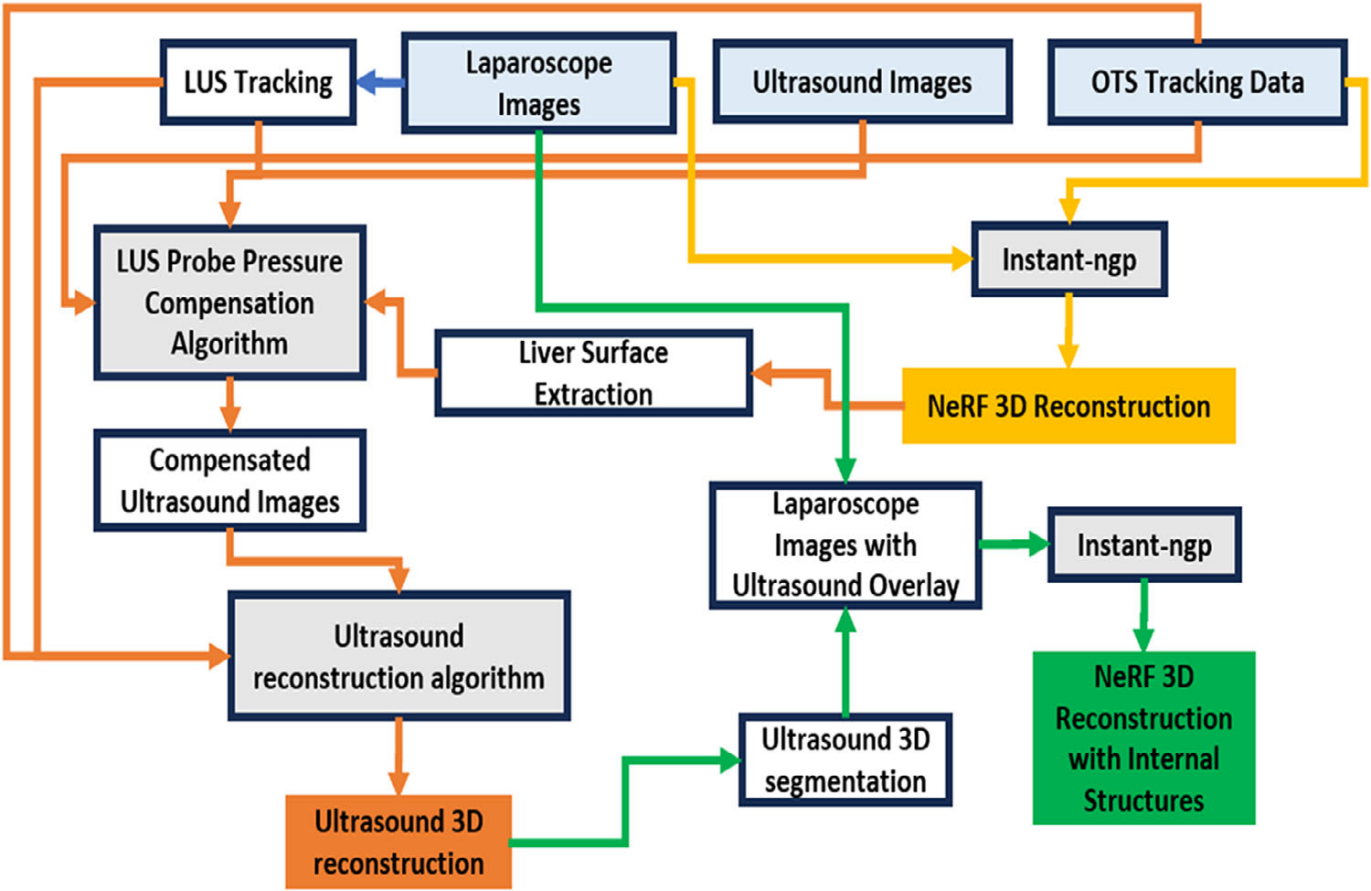
Overview of the methods.

Figure 1a shows our data acquisition setup. Our method begins with the generation of 3D reconstructions of the abdominal cavity, achieved by employing NeRFs based on optically tracked laparoscopic images (Section 2.2). To accurately track the LUS probe, we estimate its pose using tracked laparoscopic images (Section 2.1). We compensate for the pressure applied on the liver by the LUS probe, enabling the reconstruction of the liver's internal structure in 3D from the compensated ultrasound images and the corresponding LUS probe poses (Sections 2.1 and 2.3). All these reconstructions share a common coordinate system established by the OTS. Additionally, we utilize segmentations derived from the ultrasound 3D reconstructions in combination with laparoscopic images to train an NeRF that represents both the internal and external anatomy of the liver, as described in Section 2.4. The entire workflow is depicted in Figure 2.

### 2.1 LUS probe tracking and ultrasound 3D reconstruction

The tip of the LUS probe is bendable, and as it is used intra‐abdominally during surgery, it cannot be directly tracked using an OTS. Therefore, for tracking the LUS probe, we employ the hybrid approach described in Ref. [14]. A keypoints detector has been trained to identify specific points on the white pattern drawn by the manufacturer on the tip of the ultrasound probe, allowing us to estimate the pose of the probe based on the fixed geometry of these keypoints (Figure 1b). We will refer to this pattern as the Canon pattern. As the laparoscope is not static during surgery, its pose is tracked using an OTS. However, although the OTS can provide the pose of the laparoscope, it cannot directly provide the pose of the laparoscopic image. Therefore, the hand‐eye calibration matrix, $T_{I}^{L}$, which defines the transformation between the optical reflectors on the laparoscope and the coordinate system of its images, is estimated as described in Ref. [24]. The transformation between the Canon pattern and the ultrasound image, $T_{U}^{P}$, is computed as described in Ref. [14]. Equation (1) outlines the full chain of transformations required for ultrasound image tracking.

(1) $$T_{U}^{O}=T_{L}^{O}\;T_{I}^{L}\;T_{P}^{I}\;T_{U}^{P}$$

where 𝑂 represents the optical tracker, 𝑈 denotes the ultrasound image, 𝐿 is the laparoscope, 𝐼 refers to the laparoscope image, and 𝑃 is the Canon pattern on the LUS probe. Figure 1a shows our data acquisition setup and the transformation chain for the ultrasound images tracking.

The ultrasound reconstructions are performed using the algorithm described in Ref. [12], which utilizes the poses of the LUS images ($T_{U}^{O}$) and the corresponding cropped ultrasound images. However, in this work, a new method for probe pressure compensation has been developed, as described in Section 2.3.

Performing an ultrasound 3D reconstruction with this algorithm usually requires less than 10 s, and the obtained volume can be directly rendered in real time using a volume rendering software.

### 2.2 Intra‐abdominal scene reconstruction via NeRF

Instant‐NGP by Müller et al. [25] is used to perform volumetric reconstruction of the surgical scene. To bypass the challenges of camera self‐pose estimation in a featureless environment like the liver surface, we directly use the laparoscope poses provided by the OTS together with the laparoscope images from the left camera of the used stereo‐laparoscope. Generally, results of this work may be applicable to both stereo and monocular laparoscopes, which is advantageous as the latter are more commonly used in clinical practice. During the training, we utilize the extrinsic parameters optimization implemented in Instant‐NGP as a form of regularization to obtain refined poses of the laparoscope. The extracted 3D volume is directly registered into the OTS coordinate system, as the poses from the OTS are used in conjunction with the laparoscope images as input to the neural network. Performing an intra‐abdominal scene reconstruction via NeRF using tracked laparoscope images requires less than 30 s for the training and novel views can be rendered at more than 20 fps on a laptop with NVIDIA RTX A2000 GPU.

### 2.3 LUS probe pressure compensation

During ultrasound acquisitions, the probe produces deformation in the organ, which may change the position of vessels and tumors, impacting the quality and accuracy of the ultrasound 3D reconstruction. We assume the pressure applied by the probe to the organ will decay exponentially from the surface. Our approach to compensate for these deformations follows the steps described below.

#### 2.3.1 Liver surface extraction from NeRF

**FIGURE 3:**
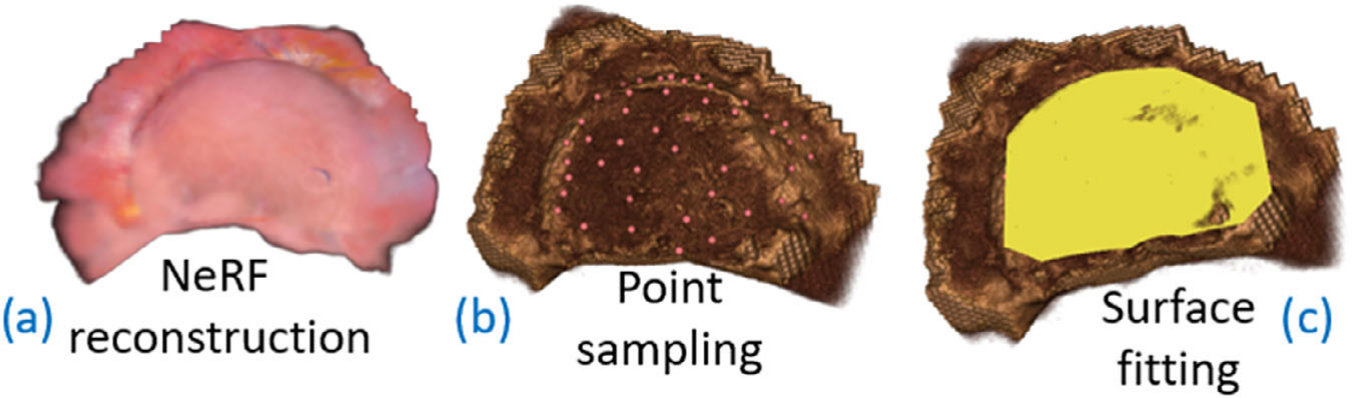
Liver surface estimation: (a) neural radiance field (NeRF) reconstruction, (b) point sampling on the surface of the generated volume, (c) surface fitting via least squares fitting.

The abdominal cavity is reconstructed from laparoscopic images and OTS laparoscope poses as described in Section 2.2 (Figure 3a). The view‐independent color and density from NeRF are resampled to a rectilinear grid and imported into 3D Slicer and visualized using volume rendering. Points on the surface of the liver are manually sampled from both 3D and MPR views (Figure 3b). A surface is then fitted to these sampled points using least squares fitting (Figure 3c). This fitted surface is used in the subsequent steps as a reference for compensating the pressure applied by the ultrasound probe. We will refer to this fitted surface as the NeRF surface.

#### 2.3.2 Liver surface extraction from ultrasound

When the ultrasound probe is in contact with the organ, the surface of the liver corresponds to the interface between the transducer and the organ. Therefore, the liver surface is extracted as the first row in each ultrasound image. We will refer to this extracted curve as the ultrasound surface.

#### 2.3.3 Calculation of displacement between surfaces

**FIGURE 4:**
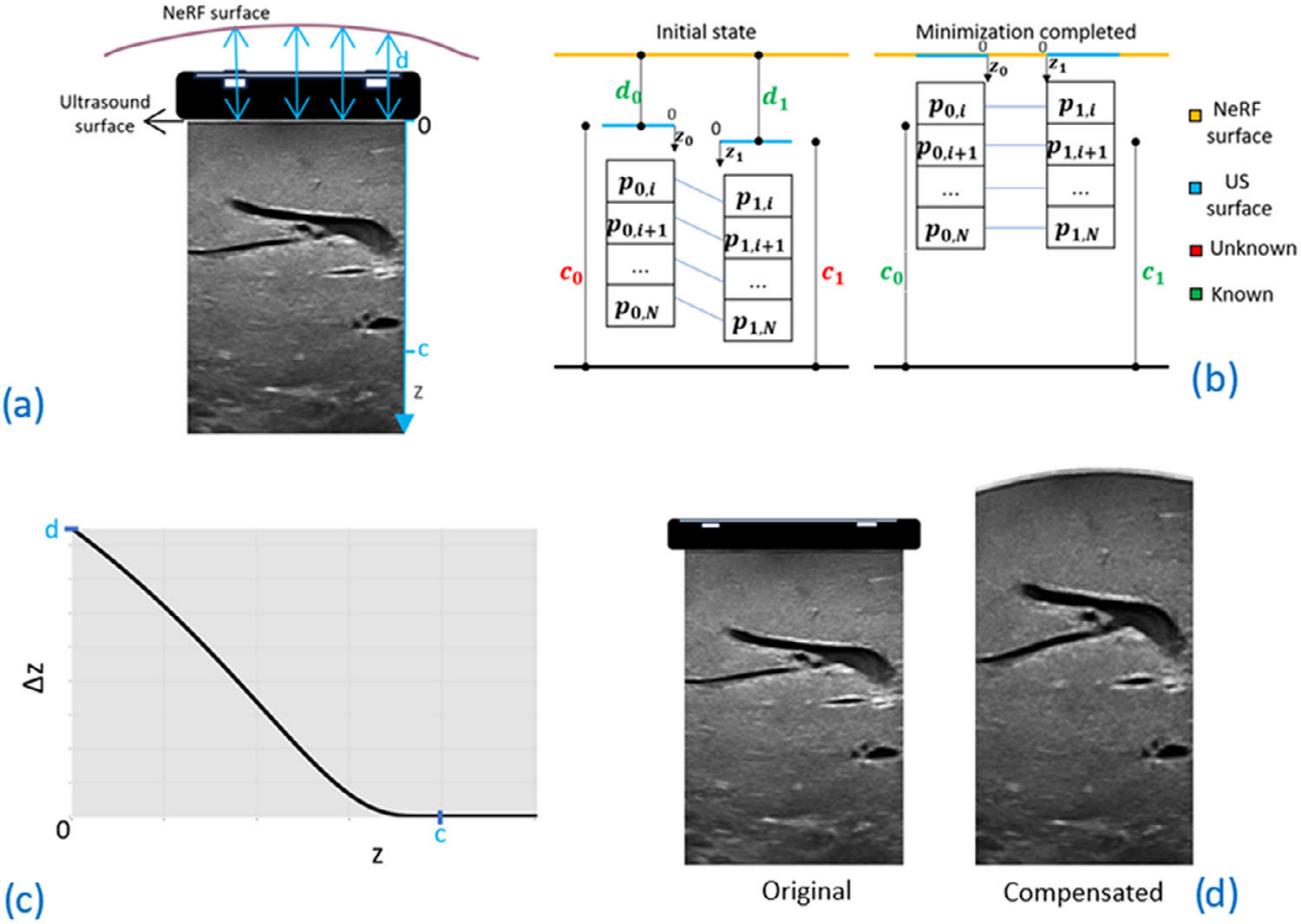
(a) Displacement between ultrasound and neural radiance field (NeRF) surfaces. (b) Schematic overview of the stress decay depth estimation. (c) Example of pressure–compensation curve. (d) Example of pressure‐compensated ultrasound image.

The vertical displacement between the NeRF surface and the ultrasound surface, denoted as 𝑑, is defined as the distance between each point on the ultrasound surface and its corresponding point on the NeRF surface (see Figure 4a).

#### 2.3.4 Stress decay depth

During ultrasound acquisition, the LUS transducer applies pressure to the liver, with the maximum pressure occurring at the surface. This pressure effect diminishes with increasing depth within the tissue. We define the stress decay depth, 𝑐, as the depth at which the residual pressure becomes negligible. The value of 𝑐 varies between ultrasound frames due to the surgeon's handling of the LUS probe, resulting in slightly different applied pressures, and due to the non‐uniform biomechanical properties of the liver. In some cases, 𝑐 may extend beyond the imaging depth, depending on the applied pressure and the tissue properties. To model the tissue deformation compensation, we use an exponential function with an exponent that varies with depth (Figure 4c). This approach is guided by the uncertainty regarding the appropriate exponent, given the unknown biomechanical properties of the liver. However, it is known that the pressure effect from the surface pressure becomes negligible at a certain depth. Therefore, we introduce 𝑐 into the exponent of Equation (2), allowing it to be estimated as described in the following section. Equation (2) describes the displacement of the ultrasound image pixels, Δ*z*, to compensate for the LUS probe pressure, for *0 ≤ z < c*:

(2) $$\Delta z=de^{-\;\frac{z}{c-z}}$$

Figure 4d shows an example of ultrasound image compensation using our algorithm.

#### 2.3.5 Stress decay depth estimation

Corresponding features are extracted from two consecutive ultrasound images using the Scale‐Invariant Feature Transform algorithm, and incorrect matches are removed using the random sample consensus method.

The stress decay depths for the two frames are estimated by determining the values 𝑐_0_ and 𝑐_1_ using Equation (3). Figure 4b shows a schematic of the minimization problem. The matched points are converted in mm by multiplying by the ultrasound scaling and are denoted as *p*_0_ and *p*_1_:

(3) $$\min_{c_{0},c_{1}}\left(\left(p_{0}-d_{0}\;e^{-\;\frac{p_{0}}{c_{0}-p_{0}}}\right)-\left(p_{1}-d_{1}\;e^{-\;\frac{p_{1}}{c_{1}-p_{1}}}\right)\right)$$

### 2.4 Integration of internal and external volumes in neural radiance fields

In our approach, ultrasound 3D reconstructions and NeRF reconstructions share a common OTS coordinate system. This alignment allows for a unified and realistic visualization of the liver. The following steps outline our process:

- *Segmentation of anatomical structures*: We first segment the anatomical structures from the 3D ultrasound reconstructions, using the 3D Slicer Segmentation module.
- *Virtual camera definition*: A virtual camera is defined with intrinsic parameters identical to those of the laparoscope used in the procedure.
- *Image rendering*: For each laparoscope image and its corresponding pose, we position the virtual camera to match the pose and render an image based on the segmented structures.
- *Image augmentation*: The rendered segmentation images are then overlaid onto the corresponding laparoscope images, creating augmented images.
- *NeRF training*: These augmented images are subsequently used to train the NeRF model.

This methodology results in a photorealistic 3D volumetric representation that simultaneously captures both the liver's surface and its internal vasculature.

## 3 EVALUATION ON CLINICAL DATA

### 3.1 Dataset

Clinical data have been acquired during liver surgery procedures and anonymized in a hospital specialized in hepato–pancreato–biliary (HPB) surgery. Data from both laparoscopic ablations and laparoscopic resections have been recorded. The datasets include videos from LUS and laparoscopic cameras, optical tracking of the laparoscope, and preoperative CTs. In addition, the laparoscopic ablation dataset includes tracking data of a patient reference fixed to the operating table, and an intraoperative CT.

### 3.2 Equipment

A 3D Rigid ENDOEYE 30° stereo laparoscope (Olympus) and a PET‐805LA LUS probe (Canon) were used during the acquisitions. A Polaris Vega XT (Northern Digital Inc) optical tracker was used to acquire the tracking data. The laparoscope was tracked by attaching to its handle a sterile optical marker from the CAS‐One Surgery kit (CAScination), whereas a sterile optical marker from the CAS‐One IR (CAScination) was used as patient reference by fixing it to the surgical table. Intraoperative CTs have been acquired using SOMATOM Definition Edge CT scanner (Siemens Healthineers) with a sliding gantry.

### 3.3 Experimental setup

The accuracy of the ultrasound 3D reconstructions is assessed comparing with GT intraoperative CT. The intraoperative CT includes the patient reference that is used to co‐register the CT volume to the optical tracker coordinate system. Five ultrasound acquisitions from a laparoscopic ablation are used to evaluate the accuracy of the reconstructions. Ultrasound volumes are reconstructed both with and without compensation for the ultrasound probe pressure based on the surface sampled from an NeRF reconstruction, to evaluate the efficacy of our method. The vessels are manually segmented from the intraoperative CT and the ultrasound reconstructions using the 3D Slicer Segmentation module. The centerlines of the vasculature are extracted using the 3D Slicer Vasculature Modeling Toolkit. The Hausdorff distance is used to evaluate the similarity between the centerlines from ultrasound and the ones from intraoperative CT. The 3D accuracy of the NeRF reconstruction is evaluated using the same laparoscopic ablation case, with intraoperative CT serving as the GT. For this evaluation, the Hausdorff distance is calculated between the liver surface segmented from the intraoperative CT and the surface generated by NeRF (Section 2.3.1). To further assess the quality of NeRF reconstructions from optically tracked laparoscope images and to demonstrate the robustness of our method on liver surgery data, we also reconstruct livers from three laparoscopic resections. However, as intraoperative CT data are not available for these three cases, we lack a GT for direct comparison. Therefore, we evaluate the rendering quality and NeRF's ability to synthesize unseen views using peak signal‐to‐noise ratio (PSNR) and structural similarity index measure (SSIM) metrics.

## 4 RESULTS

### 4.1 Ultrasound 3D reconstruction accuracy

**FIGURE 5:**
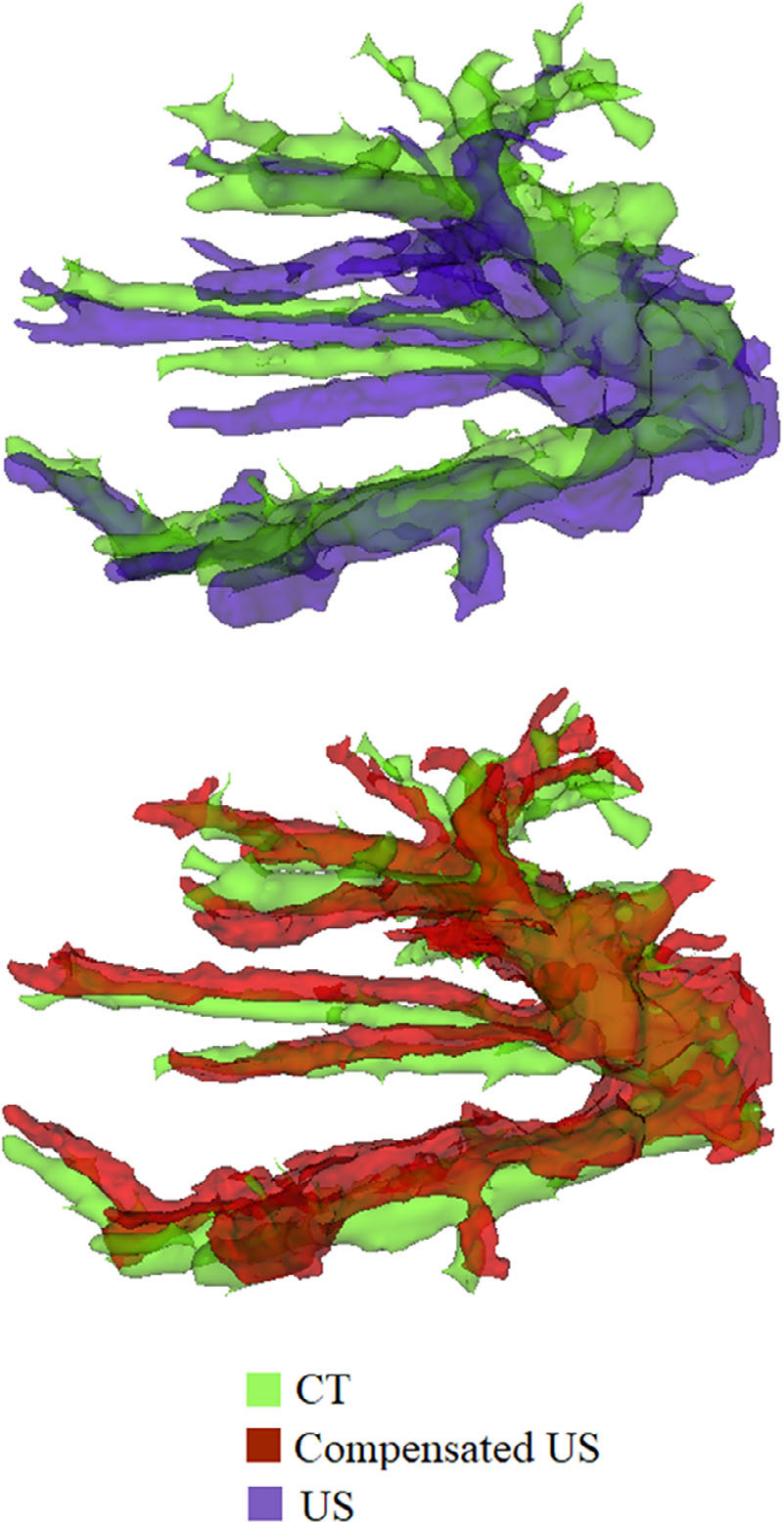
Example of comparison between segmentations from ultrasound three‐dimensional (3D) reconstruction with (bottom/red) and without (top/purple) ultrasound probe compensation. The corresponding ground truth CT region is shown in green. The same data are used for both reconstructions.

The accuracy of the ultrasound reconstruction has been evaluated over five sequences of US imaging within the same clinical laparoscopic ablation case. For each sequence, the Hausdorff distances between vessels centerlines and corresponding vessels in the GT CT have been computed. The liver vasculature is reconstructed both using our ultrasound probe pressure compensation algorithm and without correcting for it. Table 1 shows the statistics of the evaluation. A visual comparison example is also shown in Figure 5, where the segmentations of the ultrasound 3D reconstructions, from the same ultrasound sweep, with and without pressure compensation are compared to the corresponding region in the CT.

**TABLE 1:** Hausdorff distance between vessels centerlines extracted from five ultrasound 3D reconstructions and the corresponding vessels centerlines extracted from CT.

Pressure compensation	Mean (mm)	Max (mm)	Min (mm)	*Q*1 (mm)	*Q*3 (mm)	Median (mm)
No	2.6	8.0	0.0	1.5	3.6	2.2
Yes	1.6	3.3	0.2	1.0	2.2	1.5

### 4.2 Photorealistic visualization

Figure 7 shows an example of an ultrasound 3D reconstruction from the ablation dataset used for the evaluation. Figure 8a shows the NeRF reconstruction of the right lobe of the liver of the same patient. A merged visualization of both internal and external structures is shown in Figure 9, where it is possible to see an NeRF representation obtained from the same optically tracked laparoscopic images that have been augmented with a pressure‐compensated ultrasound 3D segmentation overlay. Figure 8b–d shows NeRF view synthesis of the abdominal cavity from three different laparoscopic resections with optically tracked laparoscope.

Figure 10 also includes examples of rendered images from viewpoints that were not present in the training set, with the corresponding GT images displayed for comparison. Objective results regarding the quality of the NeRF visualizations are summarized in Table 2, where PSNR and SSIM metrics are calculated across the four clinical cases on unseen laparoscopic images.

**TABLE 2:** Quantitative results obtained for video frames in the evaluation set.

	Mean	Max	Min	Q1	Q3	Median
PSNR	21.56	33.60	8.14	18.36	24.73	21.02
SSIM	0.91	0.98	0.50	0.88	0.95	0.91

*Note*: PSNR/SSIM scores are reported.

Abbreviations: PSNR, peak signal‐to‐noise ratio; SSIM, structural similarity index measure.

### 4.3 NeRF reconstruction accuracy

**FIGURE 6:**
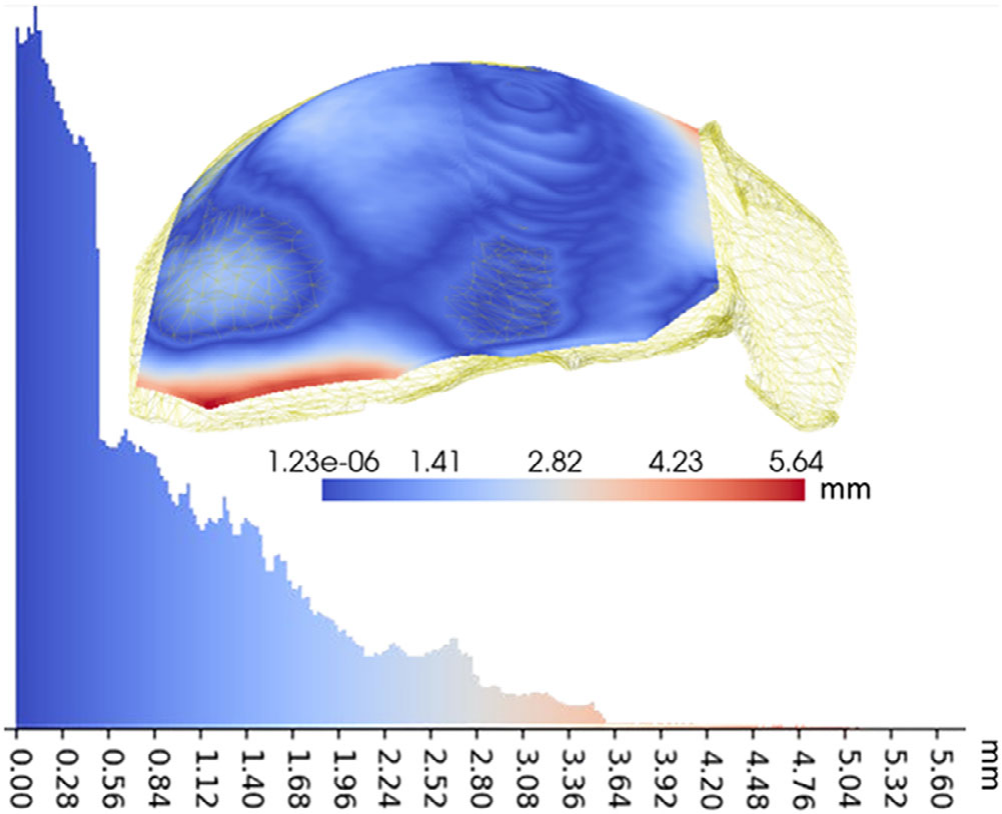
Hausdorff distances between neural radiance field (NeRF) surface and intraoperative CT.

The accuracy of the NeRF reconstruction using the laparoscopic ablation dataset is evaluated in comparison with the segmentation of the liver surface obtained from intraoperative CT. Figure 6 presents the Hausdorff distances between the two surfaces.

**FIGURE 7:**
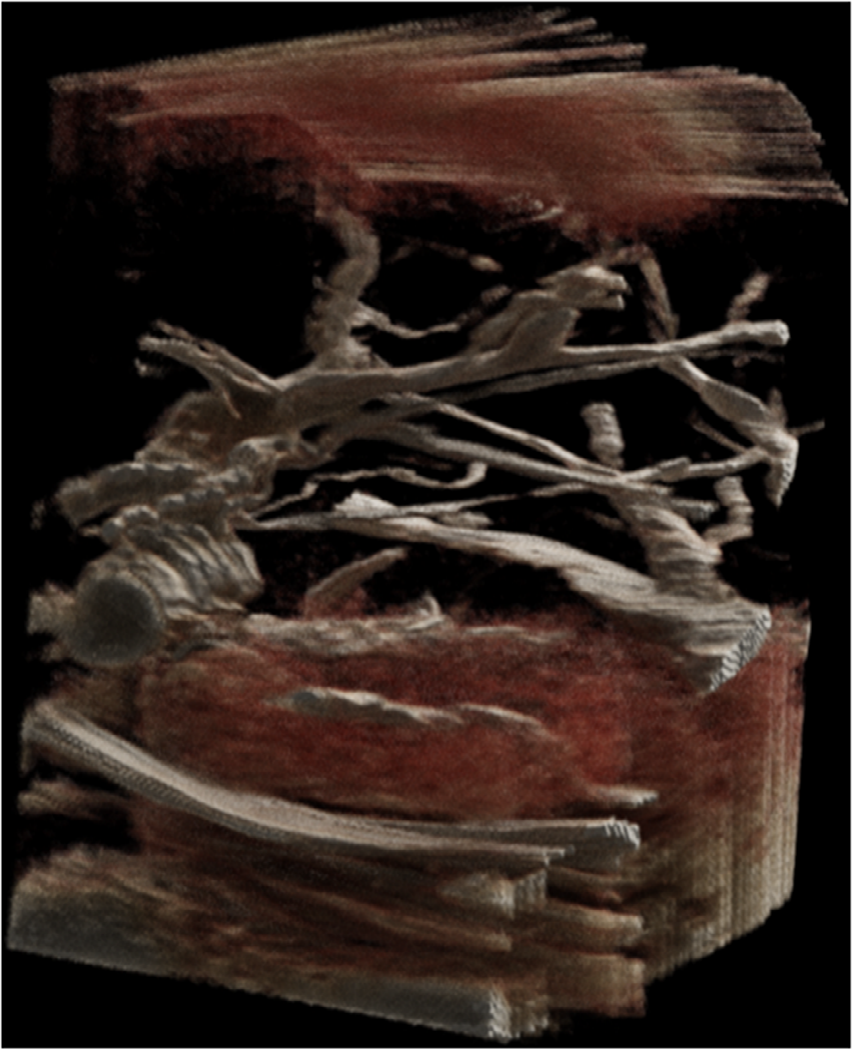
Example of visualization of an ultrasound three‐dimensional (3D) reconstruction using the Siemens Healthineers Cinematic renderer.

**FIGURE 8:**
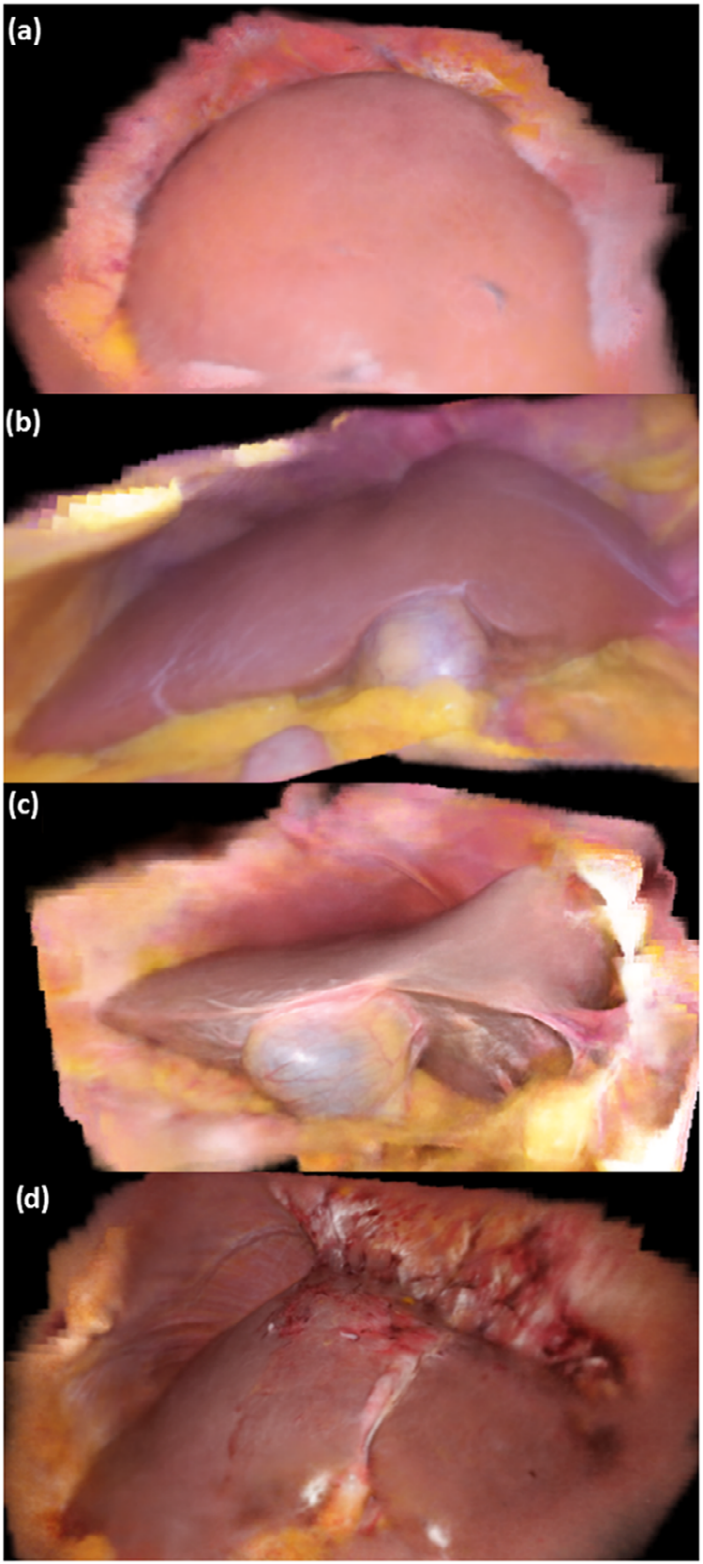
Example of neural radiance field (NeRF)‐based view synthesis from optically tracked laparoscopic images from four different patients. (a) Laparoscopic ablation. (b)‐(d) Laparoscopic resections.

**FIGURE 9:**
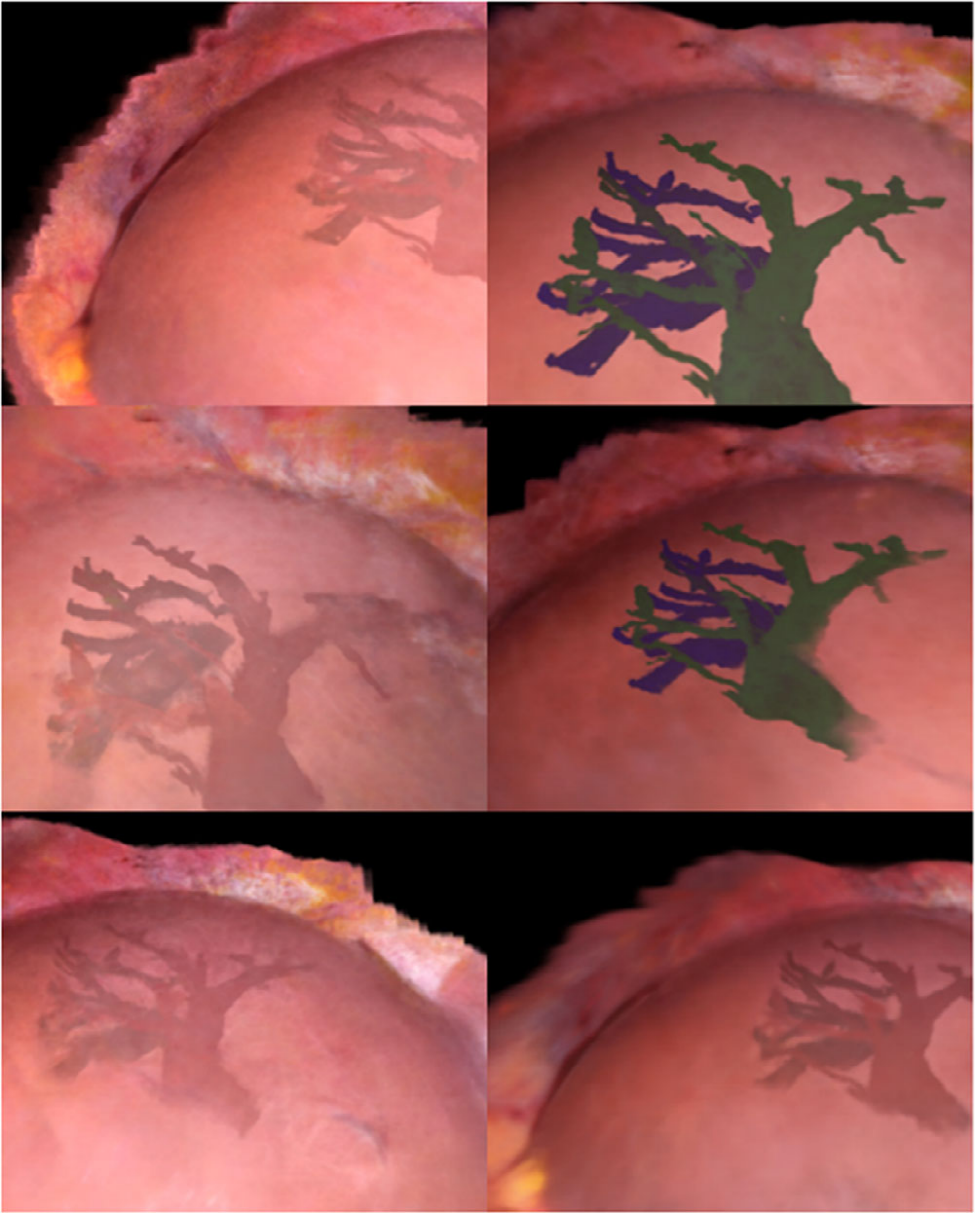
Examples of merged laparoscopic and ultrasound neural radiance field (NeRF) reconstruction from different viewpoints and different liver surface and vessels opacity.

**FIGURE 10:**
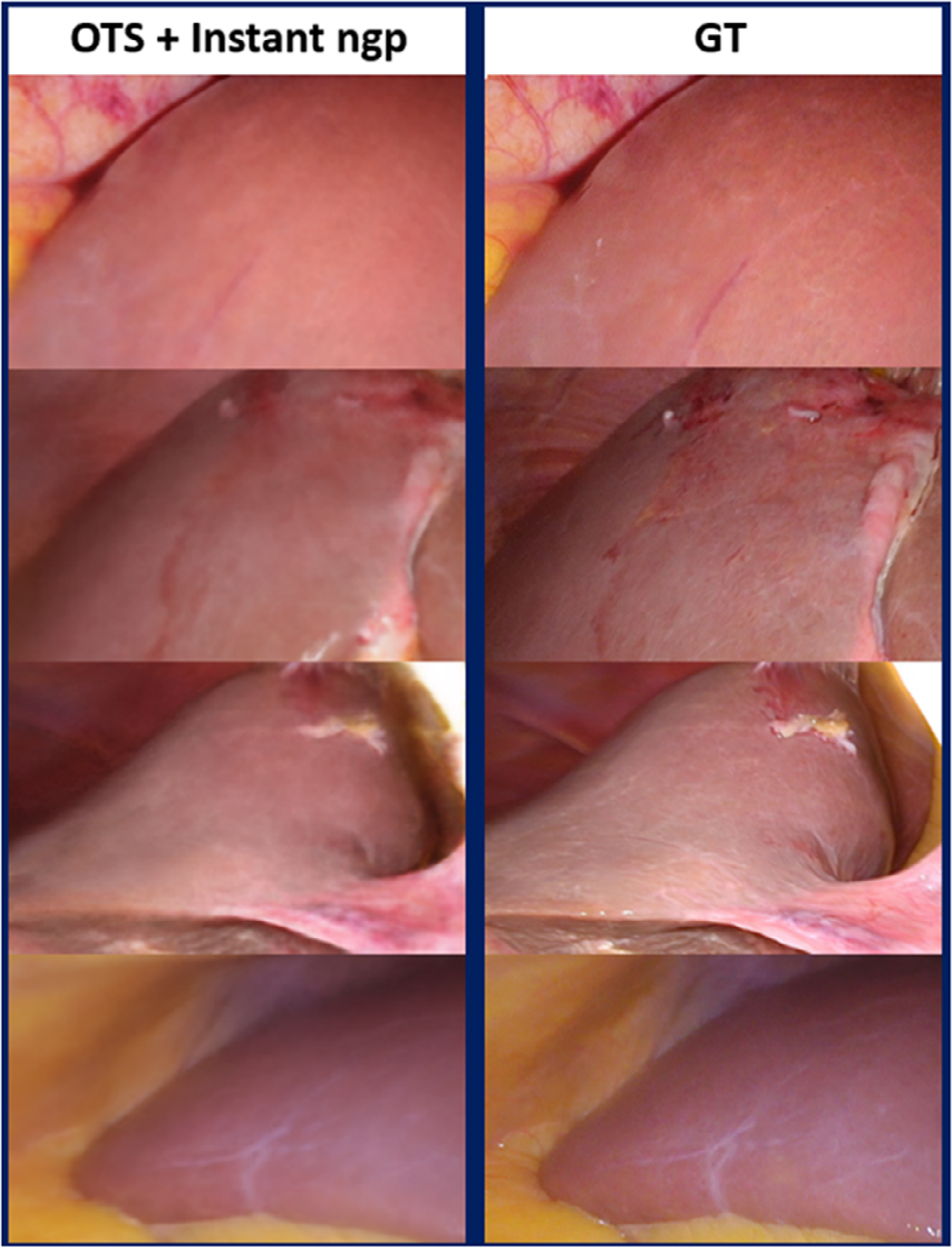
Examples of novel synthetic views generated by Instant‐NGP from unseen viewpoints (OTS + Instant‐NGP) and corresponding ground truth (GT).

## 5 DISCUSSION AND CONCLUSION

In this study, we presented a method for accurate and direct intraoperative 3D reconstruction of the liver, integrating 3D reconstructions from optically tracked laparoscopic video and ultrasound into a unified NeRF suitable for real‐time view synthesis. We developed a novel technique to compensate for the pressure applied by the ultrasound probe during image acquisition, using the surface extracted from a laparoscopic 3D reconstruction of the abdominal cavity as a reference. We show that with this method, it is possible to achieve an average navigation accuracy of 1.6 mm on the evaluated dataset with GT intraoperative CT during laparoscopy with pneumoperitoneum. Our algorithm is especially effective in correcting the deformation of the inner vessels, as visible, for example, in Figure 5.

Our results indicate that this method can provide accurate surgical navigation without significantly altering the surgical workflow, aside from the addition of optically tracking the laparoscope. In fact, we use the intraoperative CT only for accuracy evaluation, but for performing the 3D reconstructions and visualization, only laparoscopic video, LUS, and laparoscope poses from OTS are required. With a maximum navigation error of less than 4 mm, this method meets the clinical navigation accuracy requirements for laparoscopic liver surgery, according to the demands of our clinical experts, HPB surgeons with extensive academic and clinical experience.

In addition, our workflow allows to obtain an NeRF reconstruction of the liver in less 30 s from tracked laparoscope images and an ultrasound 3D reconstruction in about 10 s. The rendering speed for both reconstructions is higher than 20 fps on a laptop with an NVIDIA RTX A2000 GPU using Instant‐NGP for the rendering of the NeRF reconstructions and the Siemens Healthineers Cinematic renderer for the ultrasound reconstructions.

The obtained photorealistic visual results suggest that the combination of OTS and NeRF is a good approach for this application. The statistical values of PSNR and SSIM indicates that on average NeRF is able to synthetize realistic novel views.

Furthermore, we have proposed a novel way to directly visualize the liver in 3D intraoperatively, combining into a single‐NeRF information from the laparoscope video and the ultrasound. Motion parallax during interactive view synthesis provides an enhanced depth perception compared to a traditional ultrasound overlay over laparoscopic images. However, the 3D accuracy of this visualization must be evaluated in future works. This novel visualization requires segmentations of the vessels from the ultrasound 3D reconstruction that in this work are performed manually requiring, therefore, additional time compared to a reconstruction from original tracked laparoscope images.

Another limiting factor of this work is the manually extracted of the liver surface from the NeRF reconstruction by fitting sampled points to a surface. The accuracy evaluation of the fitted surface compared to the intraoperative CT provides errors usually lower than 2 mm, but it requires some quick manual effort. Future work will focus on developing automated solutions for this process, so that the LUS probe compensation would not require manual steps.

Additionally, our method's accuracy was evaluated using multiple intraoperative acquisitions from a single patient, due to the rarity of obtaining an intraoperative CT as GT during laparoscopic liver procedures. Typically, only laparoscopic video and LUS are used, with laparoscopic ablations being less common than the percutaneous approach. Thus, our results should be considered preliminary, and further accuracy evaluations need to be conducted on additional acquisitions from multiple patients.

With such additional evidence, this methodology has the potential to offer a novel and accessible approach to enhance intraoperative anatomical understanding by adding a new dimension to traditional laparoscopic surgery guidance, making complex procedures more accessible.

## AUTHOR CONTRIBUTIONS

**Luca Boretto**: Conceptualization; data curation; formal analysis; investigation; methodology; software; validation; visualization; writing—original draft; writing—review and editing. **Egidijus Pelanis**: Conceptualization; data curation; methodology; supervision; writing—review and editing. **Alois Regensburger**: Conceptualization; funding acquisition; methodology; project administration; supervision; writing—review and editing. **Kaloian Petkov**: Methodology; software; visualization; writing—review and editing. **Rafael Palomar**: Conceptualization; methodology; supervision; writing—review and editing. **Åsmund Avdem Fretland**: Conceptualization; data curation; methodology; resources; writing—review and editing. **Bjørn Edwin**: Conceptualization; methodology; resources; supervision; writing—review and editing. **Ole Jakob Elle**: Conceptualization; funding acquisition; methodology; project administration; supervision; writing—review and editing.

## CONFLICT OF INTEREST STATEMENT

The authors declare that L.B., A.R., and K.P. are employees of Siemens Healthineers AG or local affiliates. There are no known conflicts of interest or personal relationships that could have appeared to influence the work reported in this paper.

## Data Availability

Research data are not shared.
